# Ataxin-3 Protein and RNA Toxicity in Spinocerebellar Ataxia Type 3: Current Insights and Emerging Therapeutic Strategies

**DOI:** 10.1007/s12035-013-8596-2

**Published:** 2013-11-29

**Authors:** Melvin M. Evers, Lodewijk J. A. Toonen, Willeke M. C. van Roon-Mom

**Affiliations:** Department of Human Genetics, Leiden University Medical Center, Albinusdreef 2, 2333ZA Leiden, The Netherlands

**Keywords:** Polyglutamine disease, Spinocerebellar ataxia type 3, Machado–Joseph disease, Ataxin-3, Proteolytic cleavage, RNA toxicity

## Abstract

Ataxin-3 is a ubiquitously expressed deubiqutinating enzyme with important functions in the proteasomal protein degradation pathway and regulation of transcription. The C-terminus of the ataxin-3 protein contains a polyglutamine (PolyQ) region that, when mutationally expanded to over 52 glutamines, causes the neurodegenerative disease spinocerebellar ataxia 3 (SCA3). In spite of extensive research, the molecular mechanisms underlying the cellular toxicity resulting from mutant ataxin-3 remain elusive and no preventive treatment is currently available. It has become clear over the last decade that the hallmark intracellular ataxin-3 aggregates are likely not the main toxic entity in SCA3. Instead, the soluble PolyQ containing fragments arising from proteolytic cleavage of ataxin-3 by caspases and calpains are now regarded to be of greater influence in pathogenesis. In addition, recent evidence suggests potential involvement of a RNA toxicity component in SCA3 and other PolyQ expansion disorders, increasing the pathogenic complexity. Herein, we review the functioning of ataxin-3 and the involvement of known protein and RNA toxicity mechanisms of mutant ataxin-3 that have been discovered, as well as future opportunities for therapeutic intervention.

## Introduction

Spinocerebellar ataxia type 3 (SCA3), or Machado–Joseph disease (MJD) [1], is the most common spinocerebellar ataxia [2, 3] and the second most common polyglutamine (polyQ) disease after Huntington’s disease (HD) [4]. Similar to the other polyQ disorders, SCA3 is inherited in an autosomal dominant fashion [5], is progressively neurodegenerative and is ultimately fatal. Current therapeutic strategies are only able to provide symptomatic relief [6]. SCA3 is clinically heterogeneous, but the main feature is progressive ataxia, affecting balance, gait and speech. Other frequently described symptoms include pyramidal signs, progressive external ophthalmoplegia, dysarthria, dysphagia, rigidity, distal muscle atrophies and double vision [5, 7–9]. Neuropathological studies have detected widespread neuronal loss in the cerebellum, thalamus, midbrain, pons, medulla oblongata and spinal cord of SCA3 patients, as reviewed by Riess et al. [10].

SCA3 is caused by an expanded stretch of CAG triplets in the coding region of the *ATXN3* gene on chromosome 14q32.1, encoding the ataxin-3 protein [11]. Healthy individuals have up to 44 CAG repeats, whilst affected individuals have between 52 and 86 glutamine repeats. A repeat range from 45 to 51 is associated with incomplete penetrance of the disease [11–13]. SCA3 patients with two mutant alleles show a more severe disease phenotype than those with a single mutant allele [14]. Also, there is a clear correlation between CAG repeat size and age of onset, though CAG repeat length only accounts for approximately 50 % of the total variability in age of onset [15]. The expanded CAG repeat leads to formation of an expanded polyQ tract in the C-terminal region of the ataxin-3 protein, leading to toxic gain of function of the protein and formation of characteristic neuronal aggregates [16]. The neurotoxic properties of these aggregates are still under debate since the number of aggregates does not mirror the level of neurodegeneration or *ATXN3* CAG repeat length [17].

Despite being a monogenetic disease, SCA3 pathogenesis has proven to be complex. Extensive studies in cell and animal models over the last decade have led to the identification of several cellular processes potentially involved in SCA3 pathology. Nonetheless, much remains to be elucidated regarding the toxicity resulting from mutant ataxin-3 RNA and protein, and a more comprehensive understanding of the many cellular processes involved would be of great benefit for the development of therapeutic strategies. In this review, current knowledge on normal and mutant polyQ expanded ataxin-3 functioning, as well as the main thoughts on toxic mechanisms of mutant ataxin-3 RNA and protein and potential therapeutic strategies will be discussed.

## Ataxin-3 Protein

**Fig. 1:**
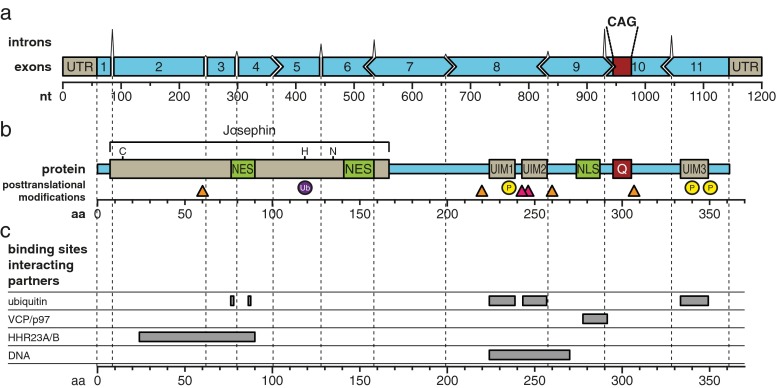
Schematic representation of the *ATXN3* gene, exon–intron structure and protein product showing protein functional domains, posttranslational modifications and binding domains of the main interacting partners. **a** The *ATXN3* gene (Ensembl transcript ID ENST00000393287) consists of 11 exons with the start codon in exon 1 and the CAG repeat in exon 10. The shape of the boxes depict the reading frame, *nt* nucleotides. The height of the introns are relative to their actual length. **b** The ataxin-3 protein consists of 361 amino acids (*aa*) with a Josephin domain in the N-terminal part that contains crucial amino acids for its isopeptidase activity [cysteine 14 (C), histidine 119 (H), and asparagine 134 (N)] and two nuclear export signals (*NES*). The C-terminal part contains three ubiquitin interacting motifs (*UIM* 1 to 3), a nuclear localisation signal (*NLS*) and the polyglutamine (*polyQ*) repeat. Specific amino acids known to undergo posttranslational modifications are indicated as follows: *yellow circles* phosphorylation (*P*), *purple eclipse* ubiquitination (Ub), *orange triangle* calpain cleavage site, *pink triangle* caspase cleavage motif. **c** Binding domains of the main interacting partners: ubiquitin; VCP/p97 valosin-containing protein, hHR23A and hHR23B human homologues of yeast protein RAD23, and DNA

The ataxin-3 protein has a molecular weight of approximately 42 kDa, depending on the size of the polyQ repeat and isoform. The CAG repeat, located in the penultimate exon, is translated into a polyQ repeat located at the C-terminus of the protein. In blood, 56 splice variants of *ATXN3* have been identified, of which 20 could potentially be translated into a functional ataxin-3 protein [18]. Of these 20 isoforms, only two isoforms that differ in their C-terminal tail have been studied extensively thus far. In this review, we will refer to the isoform of *ATXN3* most commonly expressed in brain consisting of 11 exons and translated into an ataxin-3 protein of 361 amino acids [19–21], based on a polyQ repeat length of 13 (Ensembl transcript ID ENST00000393287; Fig. 1).

## Cellular Localisation of Ataxin-3

Ataxin-3 is ubiquitously found throughout the cell and is able to translocate from the cytoplasm to the nucleus and back [20–24]. Different regions of the ataxin-3 protein influence its subcellular localisation and, although it is not yet known if ataxin-3 plays a more important role in the nucleus or the cytoplasm, enzymatically active ataxin-3 has been shown to localise to the nucleus more frequently compared to an inactive form [25]. In silico analysis predicted a nuclear localisation signal (NLS) in the proximity of the polyQ repeat at amino acid 273 to 286 (Fig. 1) [23, 26–28]. This NLS was shown to display a weak nuclear import activity in vitro [27, 28]. In contrast, however, mutating or deleting the proposed core sequence from amino acid 282 to 285 had no effect on subcellular distribution, thus questioning the importance of the ataxin-3 NLS in its cellular localisation [29, 30].

Another region that has been implicated in regulating ataxin-3 cellular localisation is the first 27 amino acids. Ataxin-3 lacking these first 27 amino acids could not be found in the nucleus, though the mechanism involved is still unknown [22]. Furthermore, ataxin-3 contains six potential nuclear export signals (NES) [26, 27], of which two (amino acid 77 to 99 and 141 to 158; Fig. 1), showed significant nuclear export activity [27]. The first NES overlaps with the ubiquitin binding site in the Josephin domain, but whether ubiquitin binding influences the function of this NES is unknown.

Nuclear localisation of ataxin-3 can also be influenced by phosphorylation events. Ataxin-3 exhibits six potential phosphorylation sites that are targets for the serine-threonine casein kinase 2 (CK2) [30] and one phosphorylation site that is a target for glycogen synthase kinase 3β (GSK 3β) [31]. Phosphorylation of serines 236, 340 and 352 by CK2 (Fig. 1) resulted in nuclear localisation of ataxin-3, whereas phosphorylation of the other serines are of less importance for subcellular localisation [30]. Next to nuclear localisation, phosphorylation of ataxin-3 was also found to be important for the stability of the protein [30].

Thus, the localisation of ataxin-3 is thought to be regulated by specific intrinsic localisation signals as well as posttranslational modifications and its cellular function likely depends on its localisation.

## Ataxin-3 and Regulation of Protein Degradation

The N-terminus of ataxin-3 contains a large Josephin domain (Fig. 1) that is known to have a low isopeptidase activity [32], implicating a role for ataxin-3 in the ubiquitin-proteasomal pathway [33]. The Josephin domain, together with the ubiquitin interacting motifs (UIMs), can either rescue proteins from degradation or stimulate breakdown by the removal of inhibitory poly-ubiquitin chains and by the regeneration of free and reusable ubiquitin [32–34]. The ubiquitin-proteasomal pathway is involved in various cellular processes such as protein degradation, endocytosis, transcriptional regulation and antigen presentation. Ubiquitination is a cascade of processes involving activating enzyme E1, transfer to ubiquitin conjugating enzymes E2 and association with ubiquitin ligases E3, resulting in addition of ubiquitins via isopeptide linkages to lysines in the targeted protein [35]. Ubiquitins can bind individually or as a poly-ubiquitin chain. Editing and removal of poly-ubiquitin chains as well as recycling of ubiquitin is critical for cellular homeostasis. Polyubiquitin chains linked through lysines 6, 11, 27, 29, 33 and 48 target proteins for proteasomal degradation. In contrast, lysine 63 or linear polyubiquitin chains have non-proteolytic functions such as activation of kinases and autophagy, where it is proposed to be involved in the biogenesis of protein inclusions [36].

Amino acid cysteine 14, histidine 119, and asparagine 134 of the Josephin domain (Fig. 1) of ataxin-3 are essential for its isopeptidase function and are highly conserved between Josephin and other ubiquitin C-terminal hydrolases and ubiquitin-specific proteases [37, 38]. The UIMs mediate selective binding to ubiquitin chains and restrict the types of chains that can be cleaved by the Josephin domain. Ataxin-3 is known to recognise poly-ubiquitin chains of four or more ubiquitins [33, 39] and binds the poly-ubiquitin linkages lysine 48, lysine 63 and mixed linkage ubiquitin chains, with preference for lysine 63-tagged ubiquitins [32, 35]. Especially the first and second UIMs are very important for binding of poly-ubiquitin chains, since mutations of leucine 229 and 249 resulted in almost abolished binding to ubiquitins [33, 40]. Additionally, within the Josephin domain, two ubiquitin binding sites were found (Fig. 1) [41]. Whilst the significance of these ubiquitin binding sites is not completely understood, mutating the first binding site eliminated binding of both lysine 48 and 63 linked ubiquitin chains to the Josephin domain of ataxin-3 [42]. The second ubiquitin binding site was shown to be important for lysine 63 tagged ubiquitins [42].

Ataxin-3 can regulate its own ubiquitination pattern [25]. Next to self-regulation, ataxin-3 can also regulate the stability and ability of the ubiquitin ligases E3 C-terminus of heat shock protein (HSP) 70-interacting protein (CHIP) [32, 43] and Parkin [44] through deubiquitination of its targets. Upon self-ubiquitination, Parkin forms a complex with conjugating enzyme E2, resulting in the addition of an isopeptide linkage to Parkin [45]. Ataxin-3 is proposed to reduce this self-ubiquitination of Parkin by removal of the isopeptide linkage [46]. Deubiquitination of CHIP by ataxin-3 was found to be only active if the poly-ubiquitin chain on target substrates reaches a critical length, preventing extra ubiquitin incorporation [47]. Contrariwise, CHIP also mono-ubiquitinates ataxin-3 at lysine 117 in the Josephin domain (Fig. 1), which directly results in enhanced isopeptidase activity of ataxin-3 [48, 49]. Interestingly, this isopeptidase activity was found to be independent of the UIMs, probably because of direct conformational changes in or near the catalytic domain [49].

Ataxin-3 has been found to bind the valosin-containing protein (VCP/p97; Fig. 1) [50, 51]. VCP/p97 has numerous functions, of which one is the regulation of misfolded endoplasmic reticulum (ER) protein degradation, a cellular process named ER-associated degradation (ERAD) [50, 52]. A potential VCP/p97 binding domain has been mapped to an arginine/lysine-rich motif just prior to the polyQ repeat [53]. The ataxin-3-VCP/p97 complex is involved in assisting targeted proteins to the proteasome [54]. Ataxin-3 is also known to interact with the human homologues of yeast protein RAD23, hHR23A and hHR23B (Fig. 1) [51]. hHR23A and hHR23B are involved in DNA repair pathways as well as the delivery of ubiquitinated substrates to the proteasome for degradation [51]. The binding site of hHR23B to ataxin-3 is located in the second ubiquitin binding site of the Josephin domain, and in concordance, hHR23B was shown to compete with ubiquitin binding [38]. Cell stress resulted in altered interactions with both VCP/p97 and HR23B, which were found mainly in the cytoplasm, although no effect on protein degradation was reported [55].

To conclude, ataxin-3 is a well-established deubiquitinating enzyme, directly regulating the ubiquitin-proteasome machinery. Next to the proteasomal degradation, ataxin-3 is also implicated to be involved in regulation of misfolded ER protein degradation.

## Ataxin-3 and Transcriptional Regulation

Besides the clear role of ataxin-3 in protein degradation, ataxin-3 has been shown to be capable of regulating the transcriptional process. Ataxin-3 is, for instance, able to repress matrix metalloproteinase-2 (MMP-2) transcription, and improved nuclear localisation of ataxin-3 through phosphorylation enhances this transcriptional repression [30]. Transcriptional regulation by ataxin-3 might arise through different mechanisms, since ataxin-3 is known to interact with numerous transcriptional regulators such as TATA box-binding protein (TBP)-associated factor 4 (TAF4) [56], cAMP response element-binding protein binding protein (CBP) [57–59], p300 [59], p300/CBP-associated factor (PCAF) [59], nuclear receptor co-repressor (NCoR1), histone deacetylase (HDAC) 3 and 6 [60, 61], forkhead box O (FOXO) transcription factor FOXO4 [62], and RAD23 [51]. Also, direct binding of ataxin-3 to DNA can likely take place through a leucine zipper motif located at amino acid 223 to 270 (Fig. 1) [61]. This basic leucine zipper motif was previously shown to bind to the GAGGAA consensus sequence in DNA [63].

Moreover, ataxin-3 can inhibit histone acetylation and repress transcription in vivo via interaction with the polyQ repeat [59]. Ataxin-3 is also postulated to regulate transcription factors by ubiquitination of CHIP transcription complexes [47]. Upon ubiquitination of transcription factors by E2 ligases, ataxin-3 is thought to be recruited to the CHIP complex, thus stimulating the transcription of the target DNA. Deubiquitination of the monoubiquitinated transcription factors by ataxin-3 would result in repression of transcription [47, 64].

These observations suggest that next to ubiquitin-proteasomal regulation of transcription, endogenous ataxin-3 might also directly interact with important transcriptional regulators.

## Is Ataxin-3 an Essential Protein for Normal Cellular Function?

Although the ataxin-3 protein has been well studied, it is still uncertain to what extent ataxin-3 is an essential protein for normal cellular functioning. In support of an essential role for ataxin-3, depletion of ataxin-3 using small-interference RNA (siRNA) in cultured non-neuronal human and mouse cells resulted in accumulation of ubiquitinated material in the cytoplasm, cytoskeletal disorganisation, loss of cell adhesion and increased cell death [65]. Contradictory evidence, however, suggests that ataxin-3 is not necessary for normal cellular functioning, since ataxin-3 knock-out in *Caenorhabditis elegans* did not alter the lifespan [66] and remarkably resulted in resistance to stress [67]. Likewise in another study local knock-down of endogenous ataxin-3 by injection of lentiviruses encoding short-hairpin RNAs (shRNAs) into the brain of wild-type rats did not show any toxicity [68]. Though ataxin-3 protein was only downregulated in the striatum and only for a 2-month period. In ataxin-3 knock-out mice, loss of ataxin-3 did not affect viability or fertility [69–71]. However, these mice did show a mild behavioural phenotype with increased anxiety, together with increased levels of ubiquitinated proteins, particularly in cells that are known to express high levels of ataxin-3 in wild-type mice [69]. Furthermore, ataxin-3 has also been proposed to serve as a neuroprotectant, since in flies expressing mutant polyQ proteins overexpression of ataxin-3 was found to alleviate neurodegeneration [72]. In contrast, double transgenic mice co-expressing mutant and wild-type ataxin-3 did not show any phenotypic improvement as compared to single transgenic SCA3 mice, suggesting that ataxin-3 may not act as neuroprotectant [73].

Whilst absence of ataxin-3 thus does not appear to be directly detrimental to cellular vitality in most studies, the subtle phenotypes observed in rodent ataxin-3 knock-out models and the fact that ataxin-3 contains several well-conserved regions amongst different species [74] may indicate that the protein is not completely dispensable.

## Altered Properties of Mutant Ataxin-3

**Fig. 2:**
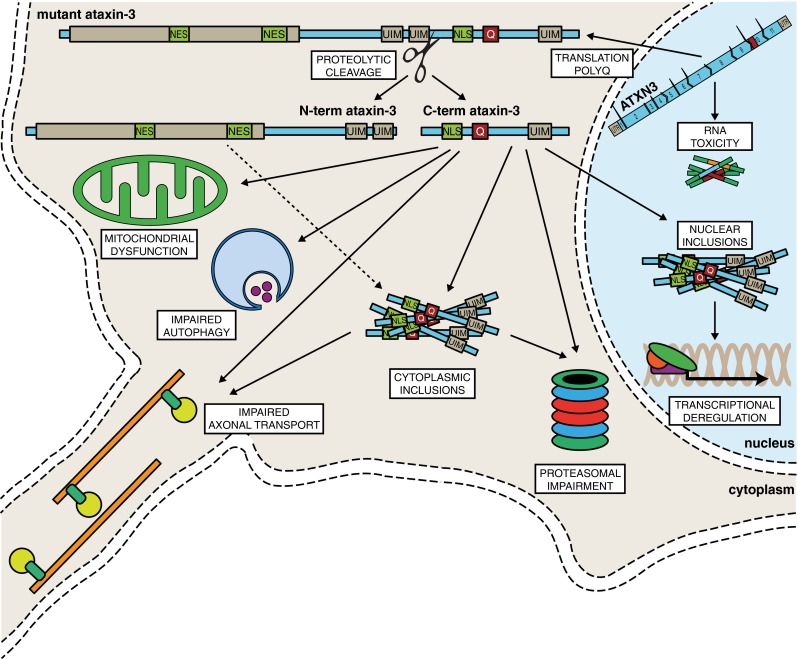
Schematic representation of cellular pathogenesis in SCA3. The *ATXN3* gene can be transcribed into mRNA containing 11 exons. The expanded CAG repeat is located in the penultimate exon and the transcript is translated into a mutant polyQ repeat containing protein. This polyQ repeat triggers conformational changes, resulting in abnormally folded mutant ataxin-3. Mutant ataxin-3 can be proteolytically cleaved, giving rise to C-terminal fragments and possibly N-terminal fragments (dashed line) that are aggregation-prone. Full length and cleaved forms of ataxin-3 form soluble monomers, oligomers or large insoluble aggregates, both in the nucleus and in the cytoplasm that cause toxicity. Other cellular disturbances resulting from mutant ataxin-3 presence involved in SCA3 pathogenesis include transcriptional deregulation, impaired autophagy, mitochondrial dysfunction, proteasomal impairment, and compromised axonal transport. Next to mutant polyQ-induced toxicity, there is likely also an RNA toxicity component involved in the disease pathogenesis

In SCA3, the expanded polyQ stretch in the C-terminus of ataxin-3 most likely leads to conformational changes of the protein, in turn resulting in altered binding properties, loss of protein function, altered subcellular localisation, aggregation, and perhaps altered proteolytic cleavage [75] (Fig. 2). Although in the past decade there has been extensive research into the SCA3 disease mechanisms, it is still not completely understood how the ataxin-3 polyQ expansion results in the observed pathology. In brain, the *ATXN3* gene expression levels were not found to be higher in the predominantly affected brain regions, suggesting that *ATXN3* gene expression levels do not directly correlate with the selective neurodegeneration seen in SCA3 patients [76]. Therefore, other alterations induced by the mutant ataxin-3 protein are most likely important in SCA3 pathogenesis as well.

## Aggregation of Mutant Ataxin-3

One of the first observations made in SCA3 patient derived brain material were the intracellular aggregates in neurons of the ventral pons and less frequently in the substantia nigra, globus pallidus, dorsal medulla and dentate nucleus [16], a feature that was reproduced in cell and animal models overexpressing mutant ataxin-3 [77–79]. Mutant ataxin-3 is known to accumulate in the cell nucleus a property that is required for in vivo toxicity [16, 20, 80]. In line with this, transgenic SCA3 mice show a decrease of soluble mutant ataxin-3 protein in the cerebellum during disease progression, whilst aggregate formation increases and the disease phenotype progresses [81]. The nuclear environment has been suggested to promote the formation of nuclear aggregates, and additional proteins, such as TBP and CBP, were found to be recruited to the aggregates in human brain [82] and SCA3 animal models [83]. Indeed, reducing nuclear localisation of mutant ataxin-3 by pharmacological inhibition of CK2 led to a reduction in nuclear inclusions [30, 31]. The intranuclear aggregates only arise when ataxin-3 contains the expanded polyQ tract [16].

Aggregation is not confined to the nucleus, however, as overexpression of mutant ataxin-3 in cultured COS-7 cells resulted in the formation of cytosolic aggregates, mainly surrounding the nucleus, suggesting a more widespread aggregation throughout the cell [22]. Likewise, widespread axonal aggregates in fiber tracts were identified in SCA3 brain tissue, similar to the axonal aggregates seen in HD [84]. These axonal aggregates potentially interfere with the axonal transport, causing impaired cell function and cell death [85, 86] (Fig. 2). Pure expanded polyQ stretches consist of monomers that are able to undergo β-sheet conformational transition and can assemble into β-sheet-rich amyloid like fibrils [87]. As polyQ regions modulate interactions between coiled-coil domains, it has been suggested that interactions of natural coiled-coil partners with the polyQ domain increase aggregation, whilst partners interacting at other regions of the mutant protein are able to diminish aggregate formation [88]. The length of the polyQ repeat of mutant ataxin-3 also determines its rate of aggregation and longer polyQ stretches are associated with increased levels of aggregation, both in vitro and in vivo [89, 90].

It is not yet entirely clear whether the aggregates observed in SCA3 brain consist of full-length ataxin-3, shorter ataxin-3 protein fragments, or perhaps a combination of the two. The aggregates in SCA3 brain material can be detected with several different antibodies, amongst which a polyQ-specific antibody, as well as an antibody binding approximately 60 amino acids N-terminal to the polyQ tract [16]. Aggregation of ataxin-3 can be induced by transfecting cells with a short ataxin-3 protein fragment with an expanded polyQ repeat [78, 91] and full-length mutant ataxin-3 is recruited into the insoluble complexes [16]. It can therefore be concluded that an expanded polyQ containing fragment can catalyse or act as a seed for aggregation of the full-length protein. Furthermore, ataxin-3 aggregates are ubiquitin-positive and contain certain proteasomal subunits, suggesting perturbation of ubiquitin-dependent proteasomal degradation in SCA3 [77] (Fig. 2). Indeed, mutant ataxin-3 undergoes ubiquitination to a similar extent as normal ataxin-3, but its half-life seems to be longer, perhaps indicating hampered degradation by the ubiquitin-proteasomal pathway [92], facilitating mutant ataxin-3 aggregation. Much is still unknown regarding the exact role and properties of protein aggregates in SCA3. However, increasing evidence suggests that the occurrence of shorter mutant ataxin-3 protein fragments is an important step leading to aggregate formation seen in SCA3.

## Proteolytic Cleavage and Formation of Toxic Ataxin-3 Fragments

A major line of thinking with regard to polyQ disorder pathogenesis termed the ‘toxic fragment hypothesis’ concerns the proteolytic cleavage of polyQ expanded protein. For mutant ataxin-3, this proteolytic cleavage is thought to lead to generation of cytotoxic and aggregation prone shorter soluble fragments containing the expanded polyQ toxic entity [91, 93–95] (Fig. 2). In a mouse model, ataxin-3 derived cleavage fragments were shown to contain expanded polyQ-containing protein fragments C-terminal of amino acid 221 [96]. Interestingly, in the two SCA3 brains tested, the ataxin-3 C-terminal fragments were enriched in disease-relevant brain structures, such as the cerebellum and substantia nigra, compared to an unaffected brain region or control brain material [96]. In subsequent studies, several caspase and calpain proteolytic enzymes were identified that could be responsible for the generation of the potentially toxic ataxin-3 fragments. These mutant ataxin-3 fragments are highly susceptible to aggregation [97], and capable of inducing both aggregation and toxicity to a larger extent than full-length mutant ataxin-3 [78, 90].

The cysteine proteases of the caspase family are associated with apoptosis and are able to cleave proteins at specific aspartate residues [98]. Caspases are implicated in the pathogenesis of several other polyQ diseases besides SCA3, though the involvement is not straightforward and varies per disease and study [99–103]. Several caspase cleavage sites are predicted for ataxin-3 and the three most relevant sites were identified to be aspartates 241, 244 and/or 248 (Fig. 1), as mutations of these residues significantly impeded caspase-1-induced ataxin-3 cleavage. In the same study, ataxin-3 cleavage lead to appearance of SDS-insoluble aggregates, and the amount of aggregates could be diminished by addition of caspase inhibitors [93]. However, the involvement of caspase-mediated cleavage of ataxin-3 in the SCA3 pathology is possibly quite limited, since incubation with purified caspases 1 and 3 only showed modest cleavage of ataxin-3. Also, under the same experimental conditions, cleavage of in vitro translated ataxin-3 was found to be less efficient when compared to cleavage of huntingtin and atrophin-1, the proteins responsible for HD and dentatorubral-pallidoluysian atrophy, respectively [104]. Furthermore, caspase 1 and 3 inhibitors did not reduce aggregate formation in a SCA3 neuronal cell line [94]. To conclude, there currently is no conclusive in vitro or in vivo evidence for an involvement of caspases in the SCA3 pathogenesis, indicating that other proteolytic enzymes are likely responsible for the formation of expanded polyQ-containing ataxin-3 protein fragments.

Increasing evidence is available for the involvement of calpains in SCA3 pathogenesis. Calpains are a family of ubiquitously expressed calcium-dependent cytosolic cysteine proteolytic enzymes [105]. Based on observed fragment sizes and antibody binding, calpain-mediated cleavage of ataxin-3 has been shown to occur most convincingly at amino acid 60, 221, and 260 (Fig. 1) [96, 97, 106, 107]. Interestingly, mutant ataxin-3 was found to be more prone to calpain-2-mediated cleavage at amino acid 260 compared to wild-type ataxin-3 and resulted in the formation of C-terminal ataxin-3 fragments highly susceptible to aggregation [97]. Inhibition of calpains resulted in reduced mutant ataxin-3 proteolysis, nuclear localisation, aggregation and alleviated toxicity in vivo [106, 107]. Conversely, enhancement of calpain activity in vivo by knocking out the calpain inhibitor calpastatin worsened the phenotype of SCA3 mice [97]. Interestingly, reduced calpastatin levels in brain tissue of SCA3 patients have been observed, indicating that depletion of calpastatin may play a role in SCA3 pathology by increasing calpain-mediated proteolysis and associated neurotoxicity [107]. Calpain-mediated ataxin-3 cleavage has also been proposed to be the determinant of neuronal specificity of pathology in SCA3. In vitro it was found that excitation-mediated calcium influx was required for ataxin-3 cleavage by calpains and subsequent formation of aggregates. This excitation-induced cleavage could only be initiated in neurons, but not in fibroblasts or glia cells [94]. Hence, in spite of some initial reports of calpain inhibitors not effecting ataxin-3 proteolysis in cell models [93, 104, 108], involvement of calpains has gained a wide base of support from both cell and animal studies [94, 97, 107].

Taken together, the mounting evidence of involvement of particularly calpains in ataxin-3 cleavage suggests an important role of these proteolytic enzymes in SCA3 pathogenesis by cleavage-induced toxicity and perhaps subsequent initiation of an aggregation cascade.

## Localisation of Mutant Ataxin-3 Protein Fragments

As outlined above, the cellular distribution of ataxin-3 is also relevant in the context of proteolytic cleavage and nuclear aggregate formation (Fig. 2). Several observations provide a strong indication that the nucleus likely is the main site of mutant ataxin-3 toxicity. An ataxin-3 construct containing only the C-terminus with the polyQ tract, but lacking the Josephin domains and UIMs, is able to aggregate in the cell nucleus or cytoplasm when coupled to a synthetic NLS or NES, respectively [29]. In the cytoplasm, however, the misfolded proteins are targets for stress-induced degradation, whereas the nuclear aggregates accumulate [29]. Similarly, in mice, the SCA3 phenotype can be exacerbated by attaching an NLS to the C-terminus of expanded ataxin-3, resulting in increased levels of nuclear aggregates and earlier death compared to the same construct without a synthetic NLS. Conversely, coupling an NES to the expanded ataxin-3 construct reduced nuclear aggregates and improved the phenotype [80]. N-terminal ataxin-3 was found to be less frequently present in SCA3 mice brain material than C-terminal fragments, which accumulated more readily and were present in the cell nucleus [107]. The cellular localisation signals are likely crucial mediators in the process of ataxin-3 aggregate localisation and possibly toxicity, as has also been suggested for SCA1 [109].

C-terminal ataxin-3 fragments arising from calpain cleavage at amino acid 260 [106] can be subject to phosphorylation at serine 340 and serine 352 within the 3rd UIM of these fragments (Fig. 1), perhaps explaining their nuclear presence and stability [30]. Other cleavage sites, such as the proposed caspase-1 cleavage around amino acid 221 and 334 [93, 96], or cleavage at amino acid 190 [110], would result in a C-terminal fragment containing only the first 2 UIMs and the polyQ tract. Preferential nuclear localisation of this fragment is again predicted due to phosphorylation of serine residues in both the UIMs, albeit to a lesser extent than a fragment containing all 3 UIMs [30].

Some indications were found that there may also be a role for cytoplasmic inclusions and even toxic N-terminal ataxin-3 fragments in SCA3 pathogenesis. Based on N-terminal fragments observed in SCA3 mouse models, it has been suggested that simultaneous cleavage at amino acid 60 and 260 may be responsible for N-terminal ataxin-3 fragments [107]. N-terminal ataxin-3 fragments can contain the Josephin domain, which by itself has an intrinsic property to aggregate into fibrils [111]. Indeed, Josephin domain self-association may play an important role in the process of aggregate formation by mutant ataxin-3 [112]. The potential role of N-terminal fragments in SCA3 was recently investigated in a mouse model expressing an ataxin-3 fragment containing the first 259 amino acids, thus including the Josephin domain, the first UIM and part of the second UIM, but lacking the polyQ tract and third UIM. The mice in this study were phenotypically normal until approximately 9 months of age, when motor coordination deficits, such as tremors and gait ataxia, reminiscent of SCA3 became apparent and the mice died prematurely. A significant degree of neuronal cytoplasmic ataxin-3 inclusions along with neuronal death was present in the cerebellum and other SCA3-relevant brain regions of these mice [113]. The preferential cytoplasmic location of the N-terminal Josephin domain containing fragment can likely be explained by the presence of the two NES [27, 28].

Taken together, there is evidence that proteolytic cleavage of mutant ataxin-3 results in the formation of both N- and C-terminal fragments. Whilst the current consensus is that the C-terminal polyQ-containing fragments localised in the nucleus are likely the more toxic entity [78, 80, 96], there might well be a role for cytosolic N-terminal ataxin-3 fragments in SCA3 pathogenesis.

## Impaired Protein Degradation in SCA3

Though ubiquitin chain proteolytic activity does not appear to vary between wild-type and mutant ataxin-3 [32], a widespread reduction of protein deubiquitination was reported in a mutant ataxin-3 cell model [32]. This potential loss of deubiquitination function in SCA3 might in part be explained by trapping of ataxin-3 and various other components of the proteasomal machinery in the large ubiquitin-rich aggregates [16, 114]. Mutant ataxin-3 binds the ERAD component VCP/p97 more efficiently than wild-type ataxin-3, probably because of conformational changes [50, 55, 115]. In spite of this stronger binding, mutant ataxin-3 seems to interfere with the degradation of target substrates [55, 116]. Additionally, N-terminal ataxin-3 fragments of 259 amino acids lacking the VCP/p97-binding domain were found to result in ER stress and impaired ER-mediated unfolded protein response when expressed in a mouse model, though ERAD component levels appeared unchanged [113].

Not only ER degradation is altered in SCA3 but also autophagy, in which the degradation of cellular components through the lysosomal machinery is impaired (Fig. 2). Aggregates of mutant ataxin-3 were found to contain molecular components involved in autophagy. For instance, beclin-1, a protein crucial in the autophagy pathway was found to be trapped in protein aggregates in SCA3 brains [117]. In a rat model overexpressing mutant ataxin-3, increased beclin-1 expression resulted in clearance of the mutant protein [117]. This observation is in accordance with impairments in autophagy seen in other neurodegenerative disorders [118], and the fact that stimulation of autophagy was found to alleviate symptoms in vivo [119].

These observations suggest that the SCA3 pathology may partly be the result of loss of function of ERAD machinery as well as compromised autophagy, together resulting in impaired protein degradation, accumulation of ubiquitinated proteins, and cellular stress.

## Mitochondrial Dysfunction in SCA3

It has been suggested that in polyQ disorders increasing oxidative stress and inability to protect against free radicals with age could lead to mitochondrial dysfunction and cell damage [120–123]. In accordance with this, a cell model overexpressing mutant ataxin-3 with 78 CAGs showed reduced antioxidant enzyme levels, increased mitochondrial DNA damage, and reduced energy supply, which indicates impaired mitochondrial function [124]. Recently, mitochondrial DNA damage was also seen in SCA3 transgenic mice expressing full-length ataxin-3 with 98 to 104 glutamines [125]. In the disease-affected pontine nuclei of these transgenic SCA3 mice, less mitochondrial DNA copies were seen, as compared to the unaffected hippocampus [125]. Additionally, less mitochondrial DNA copy numbers were observed in the mutant cells and SCA3 patient samples, implying mitochondrial DNA damage due to oxidative stress [124]. In line with this, the antioxidant enzyme superoxide dismutase is downregulated in pontine brain tissue of SCA3 patients [62], suggesting diminished antioxidant enzyme function.

Additionally, mitochondrial dysfunction was verified by the finding that the mitochondrial respiratory chain complex II activity was somewhat compromised in SCA3 [126]. As damaged mitochondria will not be able to scavenge free radicals and prevent cell energy impairment as effectively, this process may therefore further increase oxidative stress in the cell. Oxidative stress is then able to modulate vital cellular functions, potentially resulting in activation of apoptosis or excitotoxicity, two of the main causes of neuronal death [127].

Although there is not much information regarding altered mitochondrial activity in SCA3, the above described findings indicate that, like other polyQ disorders, defects in the cellular defence mechanism against oxidative stress could play a role in the pathogenesis of SCA3.

## Transcriptional Deregulation in SCA3

Since ataxin-3 displays DNA-binding properties and interacts with transcriptional regulators, transcriptional deregulation has been suggested to play a central role in the SCA3 pathogenesis [128]. In SCA3 and other polyQ disorders, transcription factors, together with polyQ proteins, are sequestered into nuclear aggregates, resulting in deregulation of their function as transcriptional co-repressor or activator [82, 83]. Transcription of genes involved in inflammatory processes, cell signalling and cell surface-associated proteins were found to be altered in SCA3 cell and mouse models, suggesting transcriptional deregulation in SCA3 [129–131]. Likewise, some corresponding proteins like MMP-2, amyloid β-protein and interleukins were found to be significantly increased in SCA3 patient brain material [129]. However, thus far no gene expression studies have been performed on SCA3 patient material to replicate above described findings.

Ataxin-3 was shown to inhibit histone acetylase activity. However, when mutated, this transcriptional repression is impaired, and increased acetylation of total histone H3 was indeed observed in mutant ataxin-3 overexpressing cells and human SCA3 brain material, resulting in an increase of transcription in SCA3 cells [61]. This transcriptional upregulation was supported by the discovery that in cells overexpressing mutant ataxin-3, MMP-2 was found upregulated [130].

Although in SCA3 the compromised transcriptional regulation currently has been less elucidated than the impaired protein degradation, the discovery of altered transcription of various genes suggests a role of transcriptional deregulation in SCA3 pathogenesis.

## RNA Toxicity in SCA3

Until recently, it was believed that polyQ disorders are solely the result of gain of toxic protein function and, to a lesser extent, loss of wild-type protein function. However, more and more observations suggest that next to protein toxicity, there is also RNA toxicity involved in the polyQ disease pathogeneses. Together with the observation that, of all polyQ disorder genes, ataxin-3 has the highest pathogenic CAG repeat threshold, there is increasing evidence that RNA toxicity also plays a role. This RNA toxicity could be involved in SCA3 pathology through alternative splicing, bidirectional transcription, involvement of the RNA interference pathway, as well as repeat-associated non-ATG-initiated translation.

## CAG Repeat Toxicity and Formation of Alternatively Spliced Transcripts

Next to gain of toxic polyQ protein function, the expanded CAG repeat itself was recently also suggested to play a role in the SCA3 pathogenesis. *Drosophila melanogaster* transgenes with a pure expanded CAG repeat in the 3′ UTR of ataxin-3 displayed progressive neural dysfunction. CAG repeats interspersed by CAA codons resulted in only a mild phenotype, indicating the importance of the expanded pure CAG repeat for the toxic phenotype [132]. However, *C. elegans* expressing an untranslated CAG repeat with 83 CAGs did not appear to have any phenotype, whereas 200 CAGs resulted in premature death [133]. Furthermore, in another *D. melanogaster* model of CAG toxicity, expressing a construct with a premature termination codon before a 93 CAG repeat, no phenotype was shown, arguing in favour of polyQ protein toxicity over RNA toxicity [134]. Therefore, the size of the CAG repeat is probably critical for the contribution of RNA toxicity from the expanded CAG repeat and since in the SCA3 patient population the repeat is between 52 and 86 CAGs, this CAG repeat toxicity may not be contributing to the observed pathology.

There is some evidence that RNA toxicity may result from stable RNA hairpin structures which can sequester RNA binding proteins, resulting in misregulation of alternative splicing. Both expanded CAG and CUG-containing RNA molecules were found to co-localise with the muscleblind-like 1 (MBNL1) splicing factor in RNA foci in both muscle and neuron nuclei [133, 135–137]. This functional inactivation of MBNL1 due to recruitment in nuclear foci resulted in misregulation of alternative splicing, which could be partly reversed by MBNL1 overexpression [138]. The potential involvement of this alternative splicing in the SCA3 pathology was shown in a *D. melanogaster* model of SCA3 were upregulation of MBNL1 was found to increase mutant ataxin-3-induced toxicity [132]. In the same study, Li et al. [132] showed that SCA3 flies with a coding CAG repeat showed a more severe phenotype than flies with CAG repeats interrupted by CAA triplets, probably because of MBNL1 upregulation in pure CAG repeat expressing flies. However, in the CTG expansion disorder myotonic dystrophy type 1 (DM1) the opposite occurs, where overexpression of MBNL1 rescues the DM1 phenotype in myoblasts [139].

Although some data is available arguing for the involvement of RNA toxicity in polyQ disorders, the exact mechanism of this expanded CAG repeat induced toxicity, perhaps through deregulation of splicing, remains elusive in SCA3 pathology.

## Bidirectional Transcription

For many genes in the genome RNA transcription occurs from both DNA strands [140, 141]. Several studies have shown that bidirectional transcription is involved in the triplet repeat disorders [142–146]. In SCA7, which is caused by a CAG repeat expansion in the *ATXN7* gene, an alternative promoter for an antisense noncoding RNA was found [146]. This SCA7 antisense noncoding transcript 1 (SCAANT1) was found to regulate sense *ATNX7* gene expression. More recently, two natural huntingtin antisense (HTTAS) transcripts were identified at the *HTT* locus [143]. One of the HTTAS contained the CTG repeat and this HTTAS was strongly reduced in HD brains. It was suggested that HTTAS negatively regulates huntingtin transcript expression [143].

For HD Like 2, a bacterial artificial chromosomes (BAC) mouse model with a pathogenic CTG repeat on the sense, and expanded CAG repeat on the antisense strand at the *Junctophilin*-*3* locus showed RNA toxicity caused by its untranslated expanded CUG repeat as well as protein toxicity by its polyQ translated expanded CAG repeat [145].

These findings suggest that in triplet repeat disorders bidirectional RNA transcription could play a role in the disease pathology by either direct CUG toxicity or deregulation of the sense transcript. Interestingly, from all nine polyQ disorders, ataxin-3 was found to have one of the highest relative amount of antisense transcriptional tags [140]. Because of the involvement of antisense transcripts in triplet repeat disorders, additional research is worthwhile to unravel the potential involvement of bidirectional transcription in SCA3.

## Involvement of the RNA Interference Pathway

Recently, short CAG repeat-containing RNAs of around 21 nucleotides, originating from mutant CAG repeat-containing RNAs were found to induce cell death [147]. The toxicity was CAG repeat dependent, since toxicity was blocked by antisense oligonucleotides against the CAG repeat sequence. These short RNAs were dicer-dependent and the mutant short CAG repeat-containing RNAs were involved in RNA interference (RNAi)-induced gene silencing of CUG repeat enclosing transcripts.

Next to small RNAs, microRNAs are also suggested to be involved in triplet repeat toxicity. MicroRNAs are small non-coding single stranded RNAs which can regulate gene expression. In DM1, the previously described splicing factor MBNL1 was found to be involved in cytoplasmic regulation of microRNAs and alteration of this microRNA processing pathway was involved in the RNA toxicity [148]. In HD, over 50 microRNAs were reported to be deregulated and could contribute to the pathogenesis in HD [149]. In a transgenic SCA3 animal, inhibition of microRNA processing strongly enhanced ataxin-3-induced neurodegeneration [150]. Furthermore, in cells overexpressing mutant ataxin-3, blocking of microRNA processing resulted in an increased toxicity [150].

Above findings advocate that both short RNAs and microRNAs contribute to the pathology seen in SCA3 and other polyQ disorders. However, the scale and importance of the involvement of RNAi pathway in the SCA3 pathogenesis remains elusive.

## Repeat-Associated non-ATG-Initiated Translation

Several reading frame variants of genes resulting in formation of alternative proteins responsible for polyQ diseases have been found, which can be categorised as (I) translational reading frame shifting and (II) repeat-associated non-ATG translation. Translational reading frame shifting was suggested using antibodies at the C-terminus in various reading frames of huntingtin and ataxin-3 [151, 152]. Next to expanded polyQ repeats, also polyalanine (polyA) stretches occur in cells derived from HD and SCA3 patients [151, 152]. These polyA stretches in the full-length ataxin-3 protein were toxic when overexpressed in *D. melanogaster* and neuronal models [153].

Repeat-associated non-ATG translation was recently proposed as a novel class of protein toxicity, in which coding RNA transcripts with mutant CAG repeats are translated in the absence of an ATG start codon [154]. This repeat-associated non-ATG translation was found in all three possible CAG repeat reading frames, resulting in the translation of proteins with polyQ, polyA, and polyserine (polyS) repeats [154]. Repeat-associated non-ATG translation together with bidirectional transcription results in seven potential reading frames translating: ATG translated polyQ proteins, non-ATG translated polyQ, polyA and polyS proteins and bidirectional non-ATG translated polyleucine (polyL), polycysteine (polyC) and polyA proteins [155]. Together with sense CAG and antisense CTG transcription, this means a total of nine possible toxic entities are potentially involved in the pathogenesis of SCA3 and other polyQ disorders.

## Potential Therapeutic Strategies

To date, only symptomatic treatment is available for polyQ disorders. For SCA3, pharmacological strategies have been developed to reduce the depression, Parkinsonian phenotype, restless leg syndrome and sleepiness [156–158]. Several clinical trials with small patient numbers have been carried out and are reviewed in Refs. [159, 160]. Clearly, there is demand for a SCA3 therapy directed at preventing or slowing the progression of neurodegeneration. For this reason, further identification of the key molecular players in the cascade leading to neuronal dysfunction and cell death in SCA3 is paramount for the development of effective therapeutic drugs. Current potential therapeutic outlooks targeting various known processes in SCA3 pathogenesis are discussed below.

## Activation of Autophagy and the Ubiquitin–Proteasome System

As silencing of mutant ataxin-3 after disease onset has been shown to rescue, to some extent, the neuropathology in a SCA3 mouse model, it can be postulated that cells are able to clear the toxic products when expression of the mutant transgene is stopped [71, 161]. For this reason, upregulation of autophagy and clearance through the ubiquitin–proteasome system may be an attractive strategy to enable cells to better cope with accumulating polyQ expanded protein. Indeed, overexpression of the beclin-1 autophagic protein led to increased clearance of mutant ataxin-3 and prevented neurodegeneration in rats [117]. Also, in SCA3 mice, upregulation of autophagy by rapamycins resulted in a reduction of aggregates and the level of soluble mutant ataxin-3, whilst the level of wild-type protein appeared unaffected [162]. Similar observations have been made in HD fly and mouse models as well [119]. Targeting autophagic pathways may therefore be a suitable treatment strategy for SCA3, and may even be beneficial after disease onset. Temsirolimus, the compound extensively used to increase autophagy in mouse models, is already in use for treatment of renal cell carcinoma and as such is known to be suitable for use in patients and might therefore be a candidate for clearance modulation based treatment of SCA3 [162]. Also, lithium chloride was shown to have therapeutic potential in a *D. melanogaster* SCA3 model. The mechanism of lithium is thought to rely on upregulation of autophagy, though anti-apoptotic effects have also been implicated [163]. A phase II/III clinical trial with lithium carbonate was recently carried out for SCA3 and showed significant reduction in the progression of gait ataxia severity [164]. However, due to the limited group size, lithium carbonate did not show a significant effect on disease progression as determined by the neurological examination score for spinocerebellar ataxia. A larger clinical trial will have to be carried out to better assess the viability of lithium as effective treatment for SCA3. Recently, H1152 was shown to be capable of ameliorating neuronal death and the neurological phenotype of SCA3 mice [165]. In this study, H1152 specifically reduced mutant ataxin-3 protein levels after intraperitoneal injection, whilst non-expanded ataxin-3 levels remained unaffected. The reduction of mutant ataxin-3 likely occurred through augmentation of the proteasome activity, thus promoting protein degradation [165]. Based on these first results, H1152 appears a promising compound for application in SCA3.

## Antioxidants

There is some evidence that defects in the cellular defence mechanism against oxidative stress could play a role in neuronal dysfunction and neurodegeneration in SCA3. Though the extent of oxidative stress involvement is not exactly known, antioxidants might be able to provide some neuroprotective effects. Free radical scavengers are able to attenuate accumulation of reactive oxygen species, and can be divided in enzymatic and non-enzymatic antioxidants. A whole range of these antioxidants have been described, providing ample opportunity for therapeutic targeting. Antioxidant-based therapies for SCA3 have been scarcely researched, but some useful lessons can be learned from the HD research field (reviewed in [166]). Notably, supplementation of the naturally occurring antioxidant creatine was shown to slow ongoing cortical atrophy in a 16-week randomised double-blind phase II clinical trial with HD patients and is therefore currently being tested in a phase III clinical trial [167]. Also, coenzyme Q10, a lipid-soluble benzoquinone possessing antioxidant potential when reduced to ubiquinol, showed some beneficial effects in HD, but did not significantly change the functional capacity score, which was the primary outcome for the trial [168]. A higher-dose clinical trial of coenzyme Q10 is currently being carried out in order to determine whether greater efficacy can be achieved. Results from these and other clinical trials with antioxidants in HD may provide interesting insights into the feasibility of antioxidant therapy for SCA3.

## Modulation of Calcium Homeostasis

Altered cellular calcium homeostasis is suggested to play a role in SCA3 pathology. Mutant ataxin-3 was found to bind and activate the intracellular calcium release channel inositol 1,4,5-trisphosphate receptor type 1 [169]. Inhibiting excessive calcium release using Dantrolene in transgenic SCA3 mice improved motor performance and prevented neuronal cell loss [169]. In neuronal cells, Dantrolene was found to have an inhibitory effect on calcium release from the ER [170]. Though multiple clinical trials using Dantrolene for other diseases show no adverse events [171, 172], studies testing the inhibition of excessive calcium release using Dantrolene in SCA3 patients have not been performed yet. Caffeine, likely operating through adenosine A_2A_ receptor antagonism [173], has shown promise in decreasing neuropathology in a SCA3 mouse model [174]. Though the mechanism of this protective effect of caffeine has not been elucidated, it can be hypothesised that the A_2A_ receptor antagonism is able to normalise glutamergic transmission [175], which might in turn prevent the calcium-dependent calpain proteolysis and aggregation of mutant ataxin-3 [94]. Irrespective of the mechanism at work, caffeine consumption may be a safe and easily implementable prophylactic strategy to delay SCA3 onset [174].

## Preventing Transcriptional Deregulation

Transcriptional deregulation resulting from mutant ataxin-3 has been observed in a SCA3 mouse model, where mRNA expression of proteins involved in signal transduction, calcium mobilisation and neuronal differentiation were found to be downregulated [131]. Given the function of the affected proteins, it might be possible that this transcriptional misregulation contributes to the pathogenic burden in SCA3. For this reason, the HDAC inhibitor sodium butyrate was tested for efficacy in SCA3 transgenic mice, and was indeed shown to reverse the mutant ataxin-3-induced histone hypoacetylation and transcriptional downregulation in the cerebellum. Also, treatment with sodium butyrate significantly improved the ataxic symptoms of these mice, thus showing good therapeutic potential [176]. In earlier mouse studies, sodium butyrate has shown good capacity in reaching the brain [177] and caused low toxicity when tested in a clinical trial for beta thalassemia [178] and a dose finding study for HD [179]. Sodium butyrate therefore seems to be a compound useful for further research with regard to efficacy for SCA3 treatment.

## Proteolytic Cleavage Inhibition

Preventing the formation of the cleavage-induced, potentially toxic, mutant ataxin-3 fragments has been suggested as a potential therapy for SCA3. Specific caspase inhibitors have been implemented in a mouse model of HD, and were indeed found to be capable of slowing disease progression [103]. Other caspase inhibitors have been investigated in clinical trials for hepatic impairment and liver transplantation, and appear to be well tolerated [180, 181]. However, caspase functioning in the brain is complex and may be important for normal brain functioning by influencing apoptosis, synaptic plasticity, dendritic development and formation of memory [182, 183]. Use of caspase inhibitors in the brain might interfere with these processes and would therefore be impractical as treatment for SCA3. Inhibition of calpains has shown good potential in reducing mutant ataxin-3 toxicity in various cell and animal models [94, 97, 106, 107]. Several types of calpain inhibitors targeting the active site of the enzyme are available, but lack specificity amongst calpain isoforms and other proteolytic enzymes [184, 185] and are therefore likely not suitable as potential therapy for SCA3.

## Preventing Aggregation

As described previously, the ataxin-3 protein aggregates are a hallmark of SCA3, and it is therefore likely that the process responsible for toxicity of mutant ataxin-3 also leads to formation of aggregates. Indeed, increased levels of certain molecular chaperones, such as HSP40 and HSP70, have been used to reduce both aggregation and toxicity of expanded polyQ tracts in several cell and mouse models, resulting in improved phenotypes [186, 187]. There are indications that these molecular chaperones increase the solubility of expanded polyQ [188, 189], which might in turn result in an increased degradation of the protein by the proteasome [190, 191], thereby alleviating toxicity. Trimethylamine N-oxide, glycerol and dimethyl sulfoxide are chemical chaperones that are able to influence protein folding and stabilise proteins in their native conformation. These chemical chaperones have been tested for their efficacy in cells overexpressing a truncated form of mutant ataxin-3, and both aggregate formation and cytotoxicity were reduced [192]. Whilst providing support for inhibition of aggregation as a therapeutic strategy, these chemicals are not fit for use in a clinical setting because they seem to be cytotoxic at concentrations required for potent suppression aggregation.

Some small molecules, such as Congo red, minocycline and chlorpromazine are useful inhibitors of aggregation in vitro, and have been tested in HD mouse models. Phenotypical rescue in these studies, however, was rather limited or absent [193–195]. Another line of research into inhibition of polyQ aggregation focusses on the use of vector encoded small antibody fragments called intrabodies. For huntingtin, intrabodies have been discovered that are capable of reducing neuropil aggregates and improve neurological symptoms in HD mouse models [196, 197]. For ataxin-3, only an aggregate-exacerbating intrabody against fibrillar polyQ proteins has been tested to date, which causes an increased cytotoxicity and cell death when combined with mutant ataxin-3 [198]. An intrabody capable of binding mutant ataxin-3 in a manner that decreases aggregation might thus be a potential candidate for relieving toxicity.

In spite of the several approaches targeting mutant ataxin-3 aggregation, no promising candidate for implementation in the clinic has been identified yet.

## Preventing Formation Mutant Ataxin-3

From a therapeutic standpoint, an advantage of monogenetic disorders such as SCA3 is that silencing of the responsible gene should result in alleviation of mutant protein toxicity. As potential gene-silencing treatment for SCA3, non-allele specific downregulation of all ataxin-3 transcripts have been tested in both wild-type and SCA3 rats [68]. This allele-nonspecific silencing of ataxin-3 in the striatum showed no adverse effects in both wild-type and SCA3 rats, and the SCA3 rats displayed locally reduced neuropathology.

However, because of the discussion regarding the importance of wild-type ataxin-3 functioning, an allele-specific downregulation targeting only the mutant allele is a more elegant and favourable approach for therapeutic application in SCA3, as has been suggested for monogenetic neurodegenerative diseases in general [199, 200]. Thus far, two strategies for allele-specific downregulation of mutant ataxin-3 have been postulated.

Firstly, allele-specific silencing was achieved through the RNAi pathway using shRNAs directed against a single nucleotide polymorphisms (SNP) unique to the mutant ataxin-3 transcript [201]. This targeted SNP at the 3′ end of the *ATXN3* gene was found to be present in over 70 % of SCA3 patients [202]. The SNP-specific shRNA was able to specifically silence mutant ataxin-3 and was found to be neuroprotective in SCA3 mouse and rat models [161, 201], thus showing good promise for clinical implementation. The first trial with nanoparticle delivered siRNA for treatment of cancer has recently shown good promise for RNAi as a potential therapeutic agent regulating gene expression [203]. Suitable delivery of RNAi therapeutics in the central nervous system, however, will first need to be optimised before a similar strategy can be attempted for SCA3.

The second approach for an allele-specific reduction of mutant ataxin-3 is aimed at targeting the expanded CAG repeat directly. Small molecules, such as antisense oligonucleotides (AONs) or peptide nucleic acids (PNAs), can in vitro achieve allele-specific silencing of mutant ataxin-3 by binding to the expanded CAG repeat, probably resulting in translational blockage of mutant ataxin-3 [204, 205]. RNAi-based approaches with abasic substitutions [206] and single-stranded silencing RNAs (ssRNAs) [207] have also been shown to be capable of allele selectively inhibiting ataxin-3 expression. A main concern regarding these CAG repeat-targeting small molecules is their specificity. There are several endogenous CAG repeat-containing transcripts, like huntingtin, TBP and other ataxins, of which downregulation might be detrimental. However, in vitro there was no evidence that endogenous CAG repeat-containing transcripts are downregulated when targeting the expanded CAG repeat directly [204], but future in vivo studies will need to provide more insight into the possible off-target effects when using CAG-specific approaches.

Next to reducing ataxin-3, an alternative oligonucleotide therapy was proposed in which the polyQ repeat is removed from the ataxin-3 protein by exon skipping [40]. With exon skipping, AONs are used that target-specific splicing signals, masking an exon in the pre-mRNA from the splicing machinery and thus resulting in exclusion of the target exon from the mRNA [208, 209]. When the correct reading frame is maintained, subsequent translation will result in formation of an internally truncated protein. Using this exon skipping technique, the toxic polyQ expansion can be removed from ataxin-3, whilst ataxin-3 expression levels remain unaltered. Exon skipping of the polyQ encoding region of ataxin-3 has recently been tested, and was shown to indeed result in formation of a shorter ataxin-3 protein lacking the polyQ repeat. The shorter ataxin-3 protein retained the main functional domains and ubiquitin binding capacity, and was not toxic in cells [40]. This same exon skipping approach was later confirmed as secondary effect of the previously described ssRNAs directed against the CAG repeat [207].

The main advantage of AONs over siRNAs is that they are efficiently taken up by neuronal cells in vivo [209], and encouraging results using AONs have been obtained in animal models for other neurodegenerative disorders like HD and amyotrophic lateral sclerosis (ALS). In HD, after intrathecal injection, AONs were shown to diffuse throughout the non-human primate brain and could be detected in neurons affected most in SCA3, which are the cerebellum, pons, midbrain and spinal cord [210]. The safety of intrathecal injection of AONs in humans was demonstrated in a phase I trial, where no serious adverse effects were observed in AON-treated ALS patients [211]. Additionally, exon skipping is currently being tested in clinical trials for treatment of Duchenne muscular dystrophy [212].

In sum, the various oligonucleotide-based approaches in development, together with the lessons learned from other neurodegenerative disorders concerning safety, potency and distribution of oligonucleotides in the brain validates the encouraging prospective of oligonucleotide-based therapeutics as treatment for SCA3.

## Final Remarks

Since the discovery of the causative gene for SCA3 in 1994, the *ATXN3* gene and resulting protein have been subject to extensive research over the last 2 decades. In this period, ataxin-3 has been established as a deubiquitinating enzyme with a function in regulating protein degradation. More recently, ataxin-3 functioning at the level of transcriptional regulation has been shown, and the extensive list of identified protein interactors hint towards yet unknown roles of ataxin-3. In parallel with the other polyQ disorders, no clear model has been established as to how polyQ expanded ataxin-3 leads to the specific symptoms of SCA3. Though the hallmark of polyQ disorders, the intracellular aggregates, are no longer regarded as the main toxic entity, the pathways leading to their formation are still being researched extensively. The involvement of proteolytic cleavage leading to toxic, potentially aggregation-prone fragments has gained increasing evidence in cell and animal models. Also, with the advance in molecular techniques, a possible role of RNA toxicity is emerging. The many pathogenic mechanisms possibly involved in SCA3 thus clearly require further research, hopefully opening new avenues for therapeutic strategies in the process. At the same time, recent advances in oligonucleotide-based therapies may well circumvent the need of a full mechanistic understanding of disease pathways, and the polyQ disorders are ideal candidates to benefit from promising new molecular therapies.

## Abbreviations

ATXN3: Ataxin-3
ER: Endoplasmic reticulum
ERAD: Endoplasmic reticulum-associated degradation
HD: Huntington’s disease
HDAC: Histone deacetylase
NES: Nuclear export signal
NLS: Nuclear localization signal
PolyQ: Polyglutamine
SCA3: Spinocerebellar ataxia type 3
UIM: Ubiquitin interacting motif
VCP/p97: Valosin-containing protein
